# Dataset on a problem of assigning activities to children, with various optimization constraints

**DOI:** 10.1016/j.dib.2019.104168

**Published:** 2019-06-21

**Authors:** Sacha Varone, Corentin Beffa

**Affiliations:** University of Applied Sciences and Arts Western Switzerland (HES-SO), HEG Genève, Switzerland

## Abstract

“Passeport Vacances”, abbreviated PV, is a set of leisure activities proposed to children to discover and enjoy during school holidays. During PV, activities are proposed several times, each one being an occurrence. This data set contains real data, collected by online registration during the summer of 2017. Children express their preferences for each available time slot. Organizers should assign activities to children by maximizing their expressed preferences, subject to several types of constraints: age limit, group size limit for each occurrence of an activity, diversification of the type of activities for each child, restrictions on costly activities, restrictions on the number of activities per period, and cost balancing. The CSV files in this data set represent the preferences of 634 children for 1121 activities over a two-week period. These data were used to develop the Morges 2017 Vacation Passport model, which is associated with the research article entitled ““Passeport Vacances”: an assignment problem with cost balancing” Beffa and Varone, 2018.

**Table:** Specifications table

Subject area	*Economics*
More specific subject area	*Operational Research/Management Science*
Type of data	*Tables in CSV format*
How data was acquired	*Online registration*
Data format	*Raw, comma separated values (CSV files)*
Experimental factors	*Participants are required to choose each day 4 activities and rank them by preference, through a formulary.*
Experimental features	*History-based data from former PV projects* *Set of activities collected by the organizers*
Data source location	*Morges, Switzerland*
Data accessibility	*Freely available with this article*
Related research article	“Passeport Vacances”: an assignment problem with cost balancing. FedCSIS Communication Papers 2018: 53–59 https://doi.org/10.15439/2018F230[1]

**Table:** 

**Value of the data** • This real-world dataset are parameters for a constrained assignment problem, used for assigning activities to children during a two-weeks discovering program. • It can be used to model and test assignation algorithms, either for educational purpose or for application testing.

## 1 Data

**Fig. 1:**
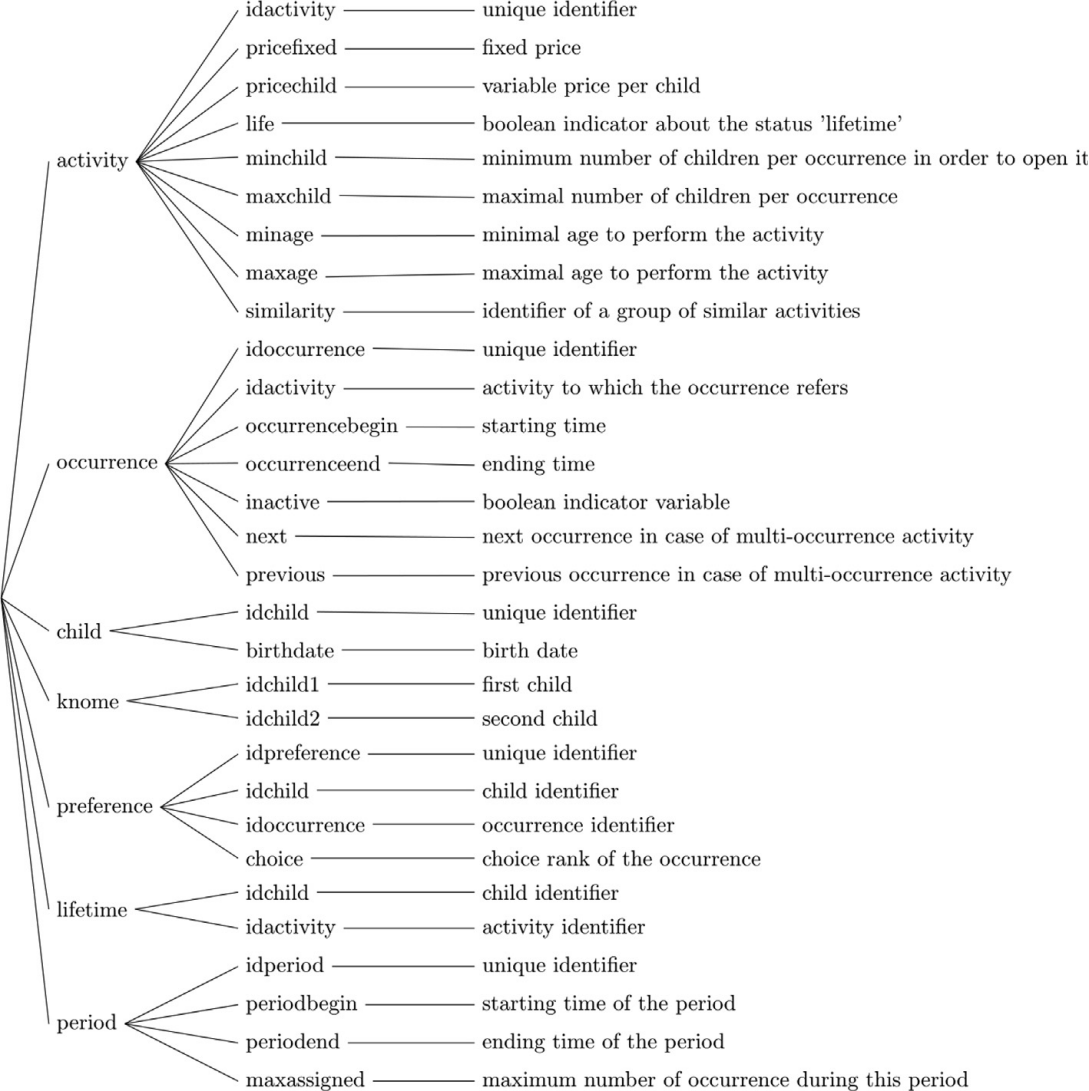
CSV's files schema.

The proposed dataset is composed of seven tables in csv format (see Fig. 1).

Table “activity” lists the data related to activities. In addition to the field dedicated to a unique identifier, the following two fields express the cost of activities: a fixed cost (*pricefixed*) and a variable cost (*pricechild*). PV is offered every year, but some costly activities can be assigned to a particular child only once (*life* is set to 1 = true). The number of children per activity is also limited (*maxchild*) and a minimal number (*minchild*) of children is required to open an activity. M*inage* and *maxage* express the minimal required age and the maximal age for each activity respectively. The *similarity* field is used to group activities together in order to restrict the number of similar activities assigned to the same child.

Activities can be organized several times during the entire PV period. Each occurrence of an activity is described in the “occurrence” table. In addition to its identifier (*idoccurrence*), its associated activity may be retrieved by the *idactivity* field. The beginning (*occurrencebegin*) and end (*occurrenceend*) of each occurrence are given. Some activities are organized over several days: the values of *next* and *previous* are respectively the identifier of the next and the previous day. For activities that require only one occurrence, their value is the identifier of the occurrence itself. The *inactive* field is for cancelled occurrences.

Information specific to children is grouped in the child's table. Each date of birth has been changed to the first day of the month and identifiers have been set from 1 to the number of children, for anonymization purposes. So the dataset can be considered as fully anonymized. Some children want to participate in the PV with friends. For such cases, the “knome” table lists all the pairs participating together, referenced by their id (*idchild1* and *idchild2*).

The preferences expressed by children for activities can be found in table “*preference*”. For each day of the PV period, a child must specify a set of up to four activities, each of which is evaluated with a priority value between 1 and 4. The occurrence concerned (*idoccurrence*) will therefore receive one of the numbers from 1 to 4 (choice). The reference to the child is *idchild* and a unique identifier for the expressed preference is *idpreference*.

Some “lifelong” activities are prohibited for children who have already obtained them during previous PVs. Therefore, all past assignments of such activities during the previous PV are kept in the “lifetime” table which is composed of the identification of a child and the identification of an activity.

Table “period” limits the number of activities assigned over a given period for each child. It is composed of an identifier (*idperiod*), a start (*periodbegin*) and end (*periodend*) time and a maximum number of assignments per child (*maxassigned*).

## 2 Experimental design, materials and methods

The PV organizers construct the *activity* and *occurrence* tables to reflect all available activities. The *lifetime* table come from previous PVs, which lists the lifelong activities formerly assigned to children. The *period* table is defined by the organizers to specify the age category.

Tables “child”, “preference” and “knome” are provided using an online registration form, which is open for nearly 4 months before the event [2].

This dataset contains 1121 activities, 634 children, which leads to 16621 ranked preferences. A linear programming model using this dataset is available in [1].
